# Discrepancies between clinical and radiological assessment of complete remission of rectal cancer after neoadjuvant radiochemotherapy

**DOI:** 10.1007/s00423-026-04047-w

**Published:** 2026-04-17

**Authors:** Katja Fechner, Maximilian Brunner, Klaus Weber, Robert Grützmann, Birgit Bittorf

**Affiliations:** 1 Department of Surgery, Friedrich-Alexander-University Erlangen-Nuremberg (FAU), Krankenhausstraße 12, D-91054 Erlangen, Germany; 2 Comprehensive Cancer Center Erlangen-EMN (CCC ER-EMN), Erlangen, Germany; 3 Comprehensive Cancer Center Alliance WERA (CCC WERA), Erlangen, Germany; 4 Bavarian Cancer Research Center (BZKF), Erlangen, Germany

**Keywords:** Rectal cancer, Diagnostics, Complete remission, Watch & wait, Radiochemotherapy

## Abstract

**Aim:**

The watch & wait concept in rectal cancer offers a promising non-operative strategy for patients who achieve a complete response following neoadjuvant radiochemotherapy (nRCT). However, accurately diagnosing clinical complete remission (cCR) based on clinical examinations, endoscopy and imaging remains a major challenge.

**Methods:**

This retrospective study included patients with rectal cancer who received nRCT at the University Hospital Erlangen between May 2022 and July 2024 (radiation: 50.4–54 Gy or 5x5 Gy; chemotherapy: 5-FU and oxaliplatin, partially as part of a total neoadjuvant therapy (TNT) concept). Patients were analyzed based on their clinical, endoscopic, and radiological findings, and classified as having either consistent or discrepant diagnostic results. Particular attention was paid to patients with discrepancies between diagnostic modalities.

**Results:**

A total of 78 patients underwent nRCT for rectal cancer during the study period. Six patients were excluded due to a second malignancy, tumor recurrence, or incomplete restaging. 72 patients were included in the final analysis. Of these, 46 patients showed consistent evidence of residual tumor during restaging, leading to surgical resection, with histopathological confirmation of residual tumor in 76% of cases. A clinical and radiological complete remission was observed in 12 of 72 patients (16.7%), four of whom experienced local regrowth. Discrepancies were noted between clinical, endoscopic and radiological findings following nRCT in 14 patients (median age 68 years, range 40–73). Of these patients, 13 had a negative MRI and one had a negative endoscopy, despite differing findings on the other modalities.

**Conclusion:**

Diagnosing complete remission after nRCT remains challenging within the context of a watch & wait strategy. Our findings suggest that pelvic MRI may overestimate the response to treatment. This is consistent with published data. Discrepancies between clinical, endoscopic, and radiologic assessments should prompt caution and emphasize the need for individualized decision-making before surgery is omitted.

## Introduction

The watch & wait concept to rectal cancer is a promising strategy for patients who achieve a complete response to neoadjuvant radiochemotherapy (nRCT). The first major investigation into the non-operative management of rectal cancer after chemoradiation was published by Habr-Gama in 2004 [1]. Since then, multiple clinical trials have investigated this new approach, confirming its feasibility and oncological safety [2–12]. The introduction of total neoadjuvant therapy (TNT), involving the addition of neoadjuvant chemotherapy, has further improved the rate of pathological complete remission (pCR) [12, 13], making non-operative management increasingly important. However, a clinically complete response (cCR) does not necessarily equate to a pathological complete response (pCR). Further studies have therefore been developed to evaluate this, including the OPRA trial and the CAO/ARO/AIO-18.1 trial (ClinicalTrials.gov identifier: NCT04246684) [12].

Nevertheless, many questions remain unanswered regarding the non-operative treatment of rectal cancer. For example, it is unclear how to identify clinical features indicating a clinical complete response, or how often this also constitutes a pathological complete response, meaning that surgery can be avoided. Risk stratification is needed to identify those patients suitable for the watch & wait concept. For example, a study by Jankowski et al. found that the watch and wait strategy should be critically questioned in patients with a primarily circumferential tumour extension or a tumour length ≥ 7 cm [14].

Guidelines recommend combining clinical, endoscopic and radiological findings for the assessment of treatment response [15–19]. Patients are categorised as having a clinical complete response (cCR), near complete clinical response (nCR) or incomplete clinical response (iCR) depending on the results [9, 12, 20, 21].

A clinical complete response (cCR) is defined as normal findings on digital rectal examination (DRE), endoscopy showing a flat white scar with telangiectasia and no ulceration or nodules, and an MRI scan showing a dark T2 signal only, few or invisible lymph nodes (< 5 mm in the short axis diameter), and absent restricted diffusion [12, 15, 20].

By contrast, a near complete response (nCR) in a digital rectal examination involves smooth induration. In endoscopy, it includes superficial ulceration, small nodules, irregular mucosa, and mild scar erythema. An MRI scan reveals a predominantly dark T2 signal with one or two foci of an intermediate T2 signal, partially regressed lymph nodes (with a short-axis diameter of ≥ 5 mm), and significant regression of restricted diffusion [12, 20, 21]. MRI imaging data suggest that diffusion-weighted imaging can predict a clinical complete response more accurately than morphology-based MRI assessment can [22–25]. The sensitivity and specificity of restaging MRI are reported in the literature as 35–100% and 31–94%, respectively [16, 26–28]. Various studies indicate that endoscopy has a sensitivity of 53–94% and a specificity of 39 − 97% [16, 29–32].

One challenge in assessing tumor responses to chemoradiation is the profound heterogeneity, even within one tumour, given that different treatment response mechanisms exist (e.g., “tumor shrinkage”, “tumor mucin pool formation” or “tumor fragmentation”) [33].

However, diagnosing complete remission using clinical examination, endoscopic and radiological criteria remains challenging.

In this study, we discuss the differences between clinical, radiological and histological findings when assessing complete remission.

## Methods

**Fig. 1:**
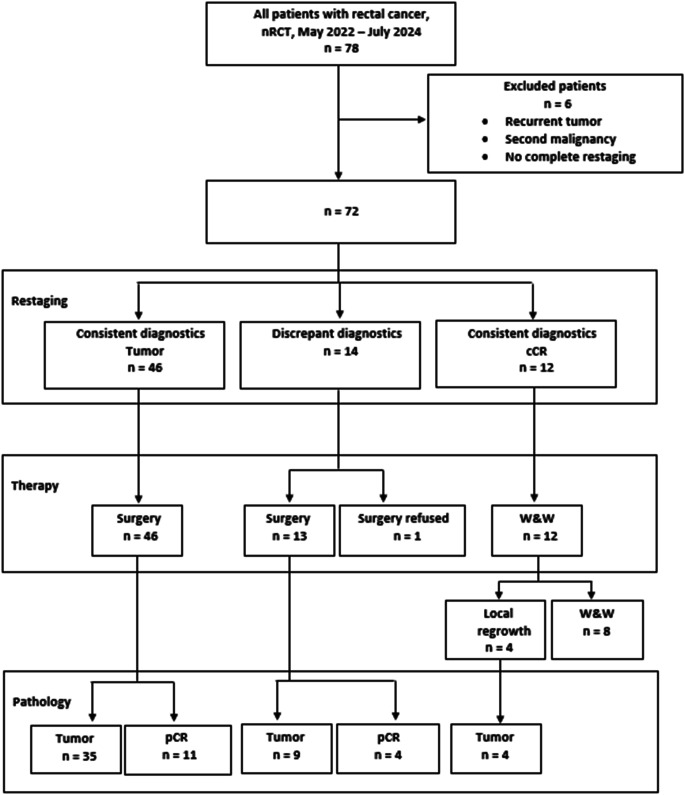
Flowchart of the study. *nRCT = neoadjuvant radiochemotherapy*,* cCR = clinical complete response*,* W&W = Watch & Wait*,* pCR = pathological complete response*

This retrospective study included 78 patients (age > 18 years) who underwent nRCT for rectal cancer at the University Hospital Erlangen between May 2022 and July 2024. Six patients with a second malignancy, a recurrent tumor, or who did not undergo complete restaging after nRCT were excluded. We analyzed the diagnostic measures (MRI and endoscopy) as consistent or discrepant in the remaining 72 patients (Fig. 1).

The data on patient characteristics and tumor classification for patients who underwent surgery and did not experience local regrowth were analyzed. The discrepancies between the clinical, endoscopic and radiological findings of patients during post-radiogenic restaging were also analyzed. All patients underwent radiation therapy with 50.4–54 Gy or 5 × 5 Gy and a chemotherapy based on 5-FU and oxaliplatin. In patients with discrepant diagnostics (*n* = 14), chemoradiation was applied as a TNT in 9 cases. Restaging was at least 8 weeks (range 8–18 weeks) after the completion of nRCT and included a digital rectal examination, endoscopy, biopsy (8/14 patients, due to mucosal proliferation in endoscopy), an MRI (diffusion-weighted and T2-weighted), tumor markers, and, if feasible, endorectal ultrasound. MRI were interpreted by at least two radiologists, and the clinical examinations were assessed by an experienced surgeon.

Statistical data analysis was performed using SPSS Statistics (version 28, IBM). Comparisons of ordinal and metric data were calculated with Mann Whitney U test. For categorial data, the chi-square test was used. Statistical significance was set at *p* < 0.05.

This study is approved by the Ethical Committee of Friedrich-Alexander-University Erlangen-Nuremberg (ethical code number 25–385-Br).

## Results

Our study analyzed a total of 72 patients, with a median age of 67 years (range 34–86 years), including 25 females. At restaging examinations, which took place at least 8 weeks (range 8 − 18 weeks) after the completion of nRCT, consistent diagnostics were observed in 58 patients (80.6%), while discrepant diagnostics were observed in 14 patients (19.4%). Table 1 shows the patients’ detailed characteristics.

**Table 1:** Patients characteristics

*n*	All patients	Consistent diagnostic	Discrepant diagnostic	*p*
	72	58 (80,6%)	14 (19,4%)	
Gender (n, %)				
female	25 (34,7%)	16 (27,6%)	9 (64,3%)	**0.014**
male	47 (65,3%)	42 (72,4%)	5 (35,7%)	
Age (years), median (range)	67 (34–86)	67 (34–86)	68 (40–73)	0.600
Tumor classification*, n	59	46	13	0.918
ypT0	15 (25,4%)	11 (23,1%)	4 (30,8%)	
ypTis/ypT1	4 (6,8%)	3 (6,5%)	1 (7,7%)	
ypT2	10 (16,9%)	7 (15,2%)	3 (23,1%)	
ypT3	28 (47,5%)	23 (50%)	5 (38,5%)	
ypT4	2 (3,4%)	2 (4,3%)	0	
Nodal status*				0.204
ypN0	49 (83,1%)	40 (87,0%)	9 (69,2%)	
ypN+	10 (16,9%)	6 (13,0%)	4 (30,8%)	

*Tumor classification: y = after neoadjuvant therapy*,* p = pathological stadium*,* T = primary tumor stadium*,* N = nodal status*, * only patients with surgery and without local regrowth

## Patients with consistent diagnostics

Of the 58 cases with consistent diagnostics, there was a clinical complete remission in 12 patients, resulting in a watch & wait approach. Of these 12 patients, four developed local regrowth.

46 patients diagnosed with residual tumour underwent surgery. Residual tumor was confirmed pathologically in 35 of these patients, and a pathological complete response was observed in 11 patients.

## Patients with discrepant diagnostics

In the assessment with discrepant diagnostics pelvic MRI showed inconspicuous findings with no evidence of tumor (negative MRI), indicating a radiological complete response in 13 patients (Table 2). One patient had unremarkable endoscopic findings (negative endoscopy).

**Table 2:** Patient characteristics and findings in patients with negative pelvic MRI (*n* = 13) and negative endoscopy (*n* = 1, Nr. 14)

Patient (number, gender, age at diagnosis)	Clinical staging prior to treatment	Neoadjuvant therapy	Digital rectal examination	MRI	Endorectal ultrasound	Endoscopy	Endoscopic biopsy	Histopathology after surgery
1, ♀, 40 years	cT3 cN2b	nRCT: 50,4 Gy, 5-FU + oxaliplatin	circular stenosis in the lower rectum	no tumor	not feasible due to stenosis	contact bleeding mucosal proliferation	necrotic material, no evidence of malignancy	ypT3b, ypN1b rectal cancer
2,♀, 64 years	cT2 cN2a	TNT: 54 Gy, 5-FU + oxaliplatin	induration in the lower rectum and the anal canal	no tumor	not feasible due to pain	contact bleeding mucosal proliferation with central ulceration	High-grade dysplasia, suspicion of invasive tumor growth	ypT2b, ypN1c rectal cancer
3,♀, 46 years	cT4a cN2a	TNT: 54 Gy, 5-FU + oxaliplatin	solid tumor in the anal canal	no tumor	hypoechoic lesion in the anal canal	mucosal proliferation with ulceration in the lower rectum and the anal canal	no biopsy performed (due to the clear findings in the rectal digital examination)	ypT3c, yN0 rectal cancer
4,♀, 62 years	cT3 cN0	TNT: 50,4 Gy, 5-FU + oxaliplatin	irregular mucosal surface of the former tumor bed	no tumor	hypoechoic lesion in former tumor bed	mucosal proliferation in the lower rectum	tubulovillous adenoma with low-grade dysplasia	ypT0, ypN0, broad-based tubular rectal adenoma with high-grade dysplasia
5,♂, 68 years	cT2 cN+	TNT: 50,4 Gy, 5-FU + oxaliplatin	normal	no tumor	not feasible due to pain	mucosal proliferation in the lower rectum and the upper anal canal	low-grade dysplasia	surgery refused
6, ♂,71 years	cT3 cN0	TNT: 50,4 Gy, 5-FU + oxaliplatin	small solid tumor in the upper anal canal	no tumor	hypoechoic suspect lesion in former tumor bed	large contact bleeding mucosal proliferation	tubulovillous adenoma with low grade dysplasia	ypT0, ypN0 rectal adenoma with high-grade dysplasia
7, ♂, 73 years	cT3 cN+	nRCT: 50,4 Gy, 5-FU + oxaliplatin	normal	no tumor	hypoechoic lesion in former tumor bed	mucosal proliferation	no dysplasia, no evidence of malignancy	ypT3d, ypN0 rectal cancer
8, ♂, 55 years	cT3 cN+	nRCT: 50,4 Gy, 5-FU + oxaliplatin	normal	no tumor	not done	small tumor	no biopsy performed	ypT2, ypN0 rectal cancer
9, ♀, 69 years	cT3 cN2a	nRCT: 50,4 Gy, 5-FU + oxaliplatin	normal	no tumor	not feasible due to stenosis	tumor stenosis in middle rectum	no biopsy performed (due to the clear findings in endoscopy)	ypT3b, ypN2b rectal cancer
10, ♀, 73 years	cT3 cN0	TNT: 54 Gy, 5-FU + oxaliplatin, mFOLFOX 6	small induration	no tumor	not feasible due to pain	ulcer	high-grade dysplasia	ypT2, ypN0 rectal cancer
11, ♀, 69 years	cT3 cN+	TNT: 50,4 Gy, 5-FU + oxaliplatin	normal	no tumor	not feasible due to stenosis	ulcerative stenosis in middle rectum	no biopsy performed (due to the clear findings in endoscopy)	ypT3, ypN1a rectal cancer
12, ♀, 67 years	cT2 cN0	TNT: 50,4 Gy, 5-FU + oxaliplatin	normal	no tumor	not done	contact bleeding mucosal proliferation	adenoma with high-grade dysplasia	ypTis, ypN0 rectal cancer
13, ♀, 73 years	cT3 cN2b	TNT: 50,4 Gy, 5-FU + oxaliplatin	circular stenosis with induration	no tumor	not feasible due to stenosis	circular tumor stenosis	no biopsy performed	ypT0, ypN0
14, ♂, 55 years	cT3 cN+	nRCT: 5 × 5 Gy, mFOLFOX6	normal	soft tissue proliferation in former tumor position	not done	white scar (complete response)	no biopsy performed	ypT0, ypN0

*nRCT = neoadjuvant radiochemotherapy*,* TNT = total neoadjuvant therapy = nRCT + neoadjuvant chemotherapy*,* 5-FU = 5-fluorouracil*,* mFOLFOX 6 = modified combination chemotherapy regimen with 5-FU + oxaliplatin + folinic acid*,* MRI = magnetic resonance imaging*

**Fig. 2:**
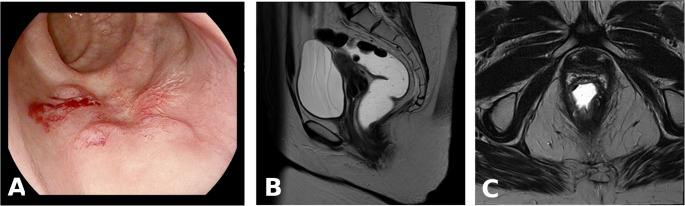
Findings of (**A**) endoscopic (irregular mucosal surface of the former tumor bed) vs. MRI image (radiological complete response (**B**) T2 sagittal, (**C**) T2 axial)

However, clinical examination revealed persistent palpable alterations in seven out of 14 patients. Endorectal ultrasound was not feasible due to pain or stenosis in 10 of the 14 patients. Four patients presented with hypoechoic changes in the former tumor bed. This included two patients with residual tumor and two patients with rectal adenoma with high-grade dysplasia, but no tumor. Mucosal abnormalities in the area of the former tumor bed were endoscopically revealed in all 13 patients with a negative MRI (Fig. 2).

Biopsies of the mucosal lesions were performed in eight cases, demonstrating low-grade dysplasia in three patients and high-grade dysplasia in three cases. In two patients, the biopsy revealed no evidence of malignancy or dysplasia.

Abdominal perineal rectal excision or low anterior resection of the rectum was recommended for all patients with discrepant diagnostics. One patient refused surgery and declined further follow-up. Of the remaining 13 patients who underwent surgery, four had a pathological complete response (ypT0), while nine had still residual tumor. Remarkably, in cases with discrepant diagnostics where MRI showed inconspicuous findings, most patients (*n* = 5; 38.5%) had locally advanced disease (ypT3) in the pathological result.

In terms of patient characteristics, the group with discrepant diagnoses had a significantly higher proportion of females (*p* = 0.014, Table 1).

## Patients who underwent surgery

The positive predictive value of endoscopy and MRI cannot be determined for all patients in our study, since 8 of the 12 patients in the watch & wait group did not undergo surgery and cannot therefore be pathologically verified. Examining only the 59 patients who underwent surgery, the positive predictive value for MRI was found to be 66%, with a sensitivity of 76%, a specificity of 20%, and an accuracy of 64%. For endoscopy, the sensitivity is 100%, the specificity 93%, the accuracy 76%, and the positive predictive value 76%, in this group of patients.

## Discussion

In 2004, Habr-Gama et al. published the first investigation of the watch & wait concept as a promising approach for patients with a complete response after neoadjuvant chemoradiotherapy (nRCT) for rectal cancer [1]. This approach is becoming increasingly popular as it preserves rectal function and quality of life, while also ensuring favourable oncological outcomes.

In our study of 72 patients undergoing nRCT for rectal cancer, discrepancies were identified in 14 cases between clinical, radiological, and histological assessments. There were significantly more females in the group with discrepant diagnostics than in the group with consistent diagnostics. The reason for this is unclear, and the literature offers no explanation for gender-specific differences in rectal cancer staging.

### Digital rectal examination

It is well established that a digital rectal examination can underestimate the extent to which rectal cancer responds to neoadjuvant therapy [34]. Guillem et al. demonstrated in a prospective analysis (*n* = 94) that the digital rectal examination underrated response in 78% of the patients after chemoradiation for rectal cancer. The overall concordance between clinical evaluation of tumor response with digital rectal examination and actual pathologic response was only 22%. The specificity of the digital rectal examination in determining a complete or near-complete pathological response was 56%, the sensitivity was 24%, and the accuracy was 49% [34]. In our patient group with discrepant diagnostics, half of the patients presented with persistent palpable alterations. It should also be noted that a digital rectal examination can only detect tumors that can be reached with the finger, which are predominantly located in the lower third of the rectum.

### Endoscopy

According to the literature, the association of endoscopic findings with clinical or pathological complete response revealed a relatively low sensitivity for endoscopic evaluation, but excellent specificity of up to 97% (Table 3).

**Table 3:** Literature of endoscopic findings in assessment of rectal cancer

Author, year	*n*	sensitivity	specificity	accuracy	PPV
Maas, 2015 [16]	50	53%	97%	n. a.	90%
Lim, 2016 [30]	106	65%	95%	89%	79%
Kawai, 2017 [29]	186	65–87%	39–78%	66–81%	n. a.
van der Sande, 2021 [32]	161	72–94%	61–85%	73–80%	63–78%
Chen, 2022 [31]	60	79%	87%	85%	65%
Williams, 2025 [35]	265	96%	33%	65%	60%
Our study (patients, who underwent surgery)	59	100%	93%	76%	76%

*PPV = positive predictive value*,* n. a. = not available*

The lower rates of specificity described by Kawai et al. [29] may be explained by the early timing of the response assessment [16, 29, 30, 32]. In the present study, mucosal abnormalities in the area of the former tumor bed were found endoscopically in all patients, though other modalities, such as MRI, showed no evidence of residual tumor. Considering these findings, endoscopic assessment appears to be more specific than other modalities, but it may overestimate residual lesions [29]. Furthermore, although endoscopy provides a good assessment of the rectal mucosa, it is limited in its ability to evaluate residual tumor in the rectal wall and mesorectum [36–38].

### Endorectal ultrasound

The overall accuracy of endorectal ultrasound for ypT staging has been reported as being quite variable in the literature, ranging from 38 to 75% [21, 39–42]. This is similar to MRI, where the challenge is also to differentiate fibrosis from residual tumor [21, 40]. Additionally, compared to MRI, endorectal ultrasound has limited applicability in cases of stenosis and in tumors in the upper rectum. This is in accordance with our experience, as endorectal ultrasound was not feasible due to pain or stenosis in 10 out of 14 patients. Four patients presented with hypoechoic changes in the former tumour bed, including two patients with residual tumour. Additionally, the accuracy of endorectal ultrasound depends heavily on the examiner [21].

### MRI

Different meta-analyses of the diagnostic performance of MRI in detecting a complete tumor response after chemoradiotherapy reveal its limited use (Table 4).

**Table 4:** Literature of MRI findings in assessment of complete tumor response after nRCT for rectal cancer

Author, year	*n*	sensitivity	specificity	accuracy	PPV
Tong, 2015 [27]	38	100%	73%	n. a.	n. a.
Maas, 2015 [16]	50	35%	94%	n. a.	75%
de Jong, 2016 (meta-analysis) [26]	790	95%	31%	75%	83%
Zager, 2024 (meta- analysis) [28]	4100	n. a.	n. a.	n. a.	70% (pooled concordance)
Williams, 2025 [35]	265	95%	23%	60%	57%
Our study (patients, who underwent surgery)	59	76%	20%	64%	66%

*nRCT = neoadjuvant radiochemotherapy*,* MRI = magnetic resonance imaging*,* PPV = positive predictive value*,* pCR = pathological complete response*,* n. a. = not available*

In a meta-analysis of 16 studies involving 790 patients, De Jong et al. reported low specificity (31%, 95% CI 14–56%) and accuracy (75%, 95% CI 72–78%) for MRI. However, sensitivity was found to be high at 95% (95% CI, 87–98%). Therefore, it was concluded that the ability of MRI to exclude complete response is superior to its ability to confirm complete response [26].

Recently, Zager et al. demonstrated in a meta-analysis of 4100 patients in 33 studies that the concordance rates between restaging MRI and pathological outcomes in patients with rectal cancer following neoadjuvant therapy are limited. The pooled concordance rate for predicting pathological complete response was 70.4% (53.6–87.1%) [28].

Nevertheless, in contrast to our experience Gollub et al. described that in 22% of the patients with a discordance between diffusion-weighted MRI and endoscopy MRI detected a tumor recurrence before endoscopy [37].

In general, MRI results seem to be biased by a substantial variability of interpretation. This is also relevant for digital rectal examination and endoscopy. A recent multireader study of MRI assessment of rectal cancer response to neoadjuvant therapy demonstrated a sensitivity of 65% for detecting complete response and specificity of 63% for detecting residual tumor with an overall accuracy of 64% and a considerable interobserver variability [43].

### Biopsies

Current international guidelines do not recommend routine biopsies of the tumor bed due to low sensitivity for detecting a complete pathological response [33, 44–47]. As tumor regression under chemoradiation does not necessarily result only in a shrinkage of the tumor mass but also in a fragmentation meaning destruction of the main tumor mass and formation of small groups of tumor cells a negative biopsy occurs in up to 54% of patients with ypT2-4 tumors [33]. In the literature, the accuracy of biopsies ranges from 36 to 75% with a sensitivity for detecting a complete pathological response ranging from 35 to 69% [33]. In our patients, biopsies were also not necessarily in accordance with the pathological result and thus proved not reliable [33, 44–47].

### Response assessment

In our study, it was surprising that a pathological ypT3 tumor was found in most cases of discrepant findings with a negative MRI. Therefore, we recommend surgery in cases where there are discrepant findings with a negative MRI and positive endoscopy results.

According to current guidelines, the assessment and follow-up of patients undergoing non-operative management for rectal cancer should include digital rectal examination, endoscopy, and a pelvic MRI [15–18]. However, there is very little information available on the accuracy of combined restaging assessments. In a prospective study of 50 patients, Maas et al. (2015) demonstrated that combining these three modalities yielded a post-test probability of a complete response of 98%. This indicates that, when all three modalities predict a complete response, this is correct in 98% of cases, with only a 2% risk of residual tumor being missed [16, 20]. Based on our experience, a positive post-test probability of 98% seems very high.

In our department of surgery, the procedure has developed to the point where we assess clinical complete remission based on the combination of digital rectal examination, endoscopy, and MRI, too. If there are any abnormalities, the clinical findings carry more weight in deciding on surgical treatment. This is also evident in the group of discrepant findings, where most patients with normal MRI results still had residual tumors. For this reason, the watch & wait concept is mainly used in cases of clinical complete remission in tumors of the lower third of the rectum, i.e., tumors that are accessible by digital rectal examination, and especially in patients with tumors in the sphincter area, for whom rectal excision would otherwise be necessary.

### Limitations

Our study has several limitations. First, it was a retrospective, single center study conducted at a cancer center specialising in multidisciplinary treatment for patients with rectal cancer. Second, the study population was small, so the results should be verified in further studies involving a larger number of cases. Third, there are differences in neoadjuvant therapy. Some patients received radiochemotherapy (50.4–54 Gy or 5 × 5 Gy), while others received TNT. Fourth, a longer follow-up is necessary to detect the outcomes in patients with watch & wait.

In conclusion, the assessment of clinical complete remission in the context of the watch & wait concept in rectal cancer remains challenging. According to the literature and to our experience, endoscopy appears to be more reliable than pelvic MRI, even with diffusion-weighted imaging, in detecting residual tumor.

Nevertheless, combining endoscopy and MRI is currently the most effective approach for evaluating responses and should be used to inform individualised treatment decisions.

## Conclusion

Diagnosing of clinical complete remission in the context of the watch & wait concept for rectal cancer remains challenging. We found that pelvic MRI largely overestimated the clinical response to nRCT. Discrepancies between clinical, endoscopic, and imaging findings should prompt great caution and an individual re-evaluation of the non-operative approach.

## Data Availability

The datasets generated during the current study are available from the corresponding author on reasonable request, but are not public due to privacy restrictions, as they were obtained from medical records.
